# Agricultural R&D, technology and productivity

**DOI:** 10.1098/rstb.2010.0140

**Published:** 2010-09-27

**Authors:** J. Piesse, C. Thirtle

**Affiliations:** 1 Business School, Bournemouth University, 89 Holdenhurst Road, Bournemouth BH8 8EB, UK; 2 Department of Agricultural Economics, University of Stellenbosch, Private Bag X1, Matieland 7602, Republic of South Africa; 3 Centre for Environmental Policy, Imperial College London, Exhibition Road, London SW7 2AZ, UK

**Keywords:** agricultural R&D, technology, productivity growth

## Abstract

The relationships between basic and applied agricultural R&D, developed and developing country R&D and between R&D, extension, technology and productivity growth are outlined. The declining growth rates of public R&D expenditures are related to output growth and crop yields, where growth rates have also fallen, especially in the developed countries. However, growth in output value per hectare has not declined in the developing countries and labour productivity growth has increased except in the EU. Total factor productivity has generally increased, however it is measured. The public sector share of R&D expenditures has fallen and there has been rapid concentration in the private sector, where six multinationals now dominate. These companies are accumulating intellectual property to an extent that the public and international institutions are disadvantaged. This represents a threat to the global commons in agricultural technology on which the green revolution has depended. Estimates of the increased R&D expenditures needed to feed 9 billion people by 2050 and how these should be targeted, especially by the Consultative Group on International Agricultural Research (CGIAR), show that the amounts are feasible and that targeting sub-Saharan Africa (SSA) and South Asia can best increase output growth and reduce poverty. Lack of income growth in SSA is seen as the most insoluble problem.

## 1. Introduction

Since the Second World War increasing agricultural productivity allowed food output to more than keep pace with demand, so that the long-term trend in prices was downward. This resulted from the application of science to agriculture on the biological side, first in the developed countries (DCs), but with a considerable public sector interest that ensured the transmission to less developed countries (LDCs). It also led to the success of the green revolution. But since 2008, rising food prices have swept away the apathy that had caused the neglect of agriculture over the last two decades (Piesse & Thirtle 2009; see the electronic supplementary material, table 1 and glossary).

**Table 1.** Acronyms.

acronym	definition
Bt	*Bacillus thuringiensis*
CGIAR	Consultative Group on International Agricultural Research
CIMMYT	International Maize and Wheat Improvement Centre (English translation)
DCs	developed countries
FSU	former Soviet Union
GDP	gross domestic product
GM	genetically modified
GPG	global public good
IPRs	intellectual property rights
IRR	internal rate of return
LAC	Latin America and Caribbean
LDCs	less developed countries
MENA	Middle East and North Africa
MNCs	multinational companies
MTA	material transfer agreement
NARS	national agricultural research systems
NIEs	newly industrializing economies
R&D	research and development
ROR	rate of return
SSA	sub-Saharan Africa
TFP	total factor productivity
TRIPS	agreement on trade related aspects of intellectual property rights
WDR	World Development Report
WEMA	water-efficient maize for Africa
WTO	World Trade Organization

Thus, §2 outlines the way in which R&D generates new technologies and extension transmits them to farmers, increasing productivity domestically and internationally. This encompasses the role of the DCs in generating basic advances that spill over to the developing counties and the role of extension services in the dissemination of technology.

Section 3 addresses international comparisons of R&D expenditures and different measures of productivity growth. Increases in yields are crucial to output growth, but labour productivity growth is dominant in the DCs and is most closely correlated with wages and incomes in the LDCs. Labour productivity increases as human labour is replaced first by animal and then mechanical power, which has been driven predominantly by private sector R&D. These partial measures do not take account of this substitution process, but total factor productivity (TFP) indices do. TFP also distinguishes between technical progress, efficiency change and input intensification, so TFP growth has different implications, according to its cause.

Section 4 explains the effects of the expansion of private R&D, biotechnology and patents, which have together led to a rapid concentration in the sector, so that there are now less than half a dozen major producers of new varieties. This represents a threat to the public good nature of agricultural technology on which the green revolution was based. Section 5 reports estimates of the levels of agricultural investment that would be needed to end hunger by 2025. The conclusion summarizes and evaluates the evidence.

## 2. R&D, extension and productivity growth

The two simplest productivity measures are partial: yield and the average product of labour. New data from Alston *et al*. (2008) show that for the US, between 1866 and 2007 average yields of maize increased by a factor of 6 and wheat yields by a factor of 3.5. In 2002, US agricultural production was more than five times its 1910 level. The increase in output from 1910 to 2002 was 1.82 per cent per year, achieved with only a 0.36 per cent per year increase in aggregate inputs. Thus, from 1911 to 2002, yields increased by a factor of 4.4, labour productivity by a factor of 15.3 and TFP by a factor of 4.1. Similarly, by the early 1980s in the UK, the labour input required to produce crops like potatoes, sugar beet, wheat and barley was only one-tenth the 1930 level, and over the same period wheat yields increased by a factor of 3 (Grigg 1989). From the Second World War to the early 1980s, tractor horsepower increased more than ten-fold and nitrogen fertilizer application grew by a factor of 6 (Holderness 1985).

These achievements required massive and sustained expenditures on R&D. The US expenditures are recorded by Huffman & Evenson (1992) and those for the UK by Thirtle *et al*. (1997). The review of the literature on the returns to R&D by Alston *et al*. (2000) leaves no doubt that R&D expenditures have led to these productivity gains. For the developing countries, the average internal rate of return (IRR) is 43 per cent. Evenson (2001) similarly reviewed a large number of studies and found the IRR to be above 40 per cent but with a large variance. These results are generally taken to be evidence of under-investment in agricultural R&D and Evenson shows that the same is true for extension.

Much of the improvement in plant materials was the work of the public sector, while mechanical innovations have been mostly attributable to private R&D. The diffusion of both biological and mechanical innovations takes many years, so there is a lag between the R&D expenditures and the productivity gains at the farm level that can be 25 to 40 years. R&D produces yield gains at the trial plot level, which then require expenditures on extension to take them to the farmer. Then, since more educated farmers are generally better at screening and adapting new technologies, farmer education plays a role. There is also good evidence that spillovers between research jurisdictions are as important as direct benefits within countries (Schimmelpfennig & Thirtle 1999). The relationship between public and private R&D has been less studied, but it seems probable that the two are complements rather than substitutes (Thirtle *et al*. 2004). The relationship between basic and applied research was pioneered by Evenson *et al*. (1979) and the lags between basic and applied research and diffusion are modelled in Thirtle *et al*. (1998).

The international transmission of productivity-enhancing technologies depends on the rate at which new technology becomes available, the extent to which it is allowed and encouraged to spill over into other jurisdictions and the capacity of the recipient countries to identify, customize and diffuse it. Hayami & Ruttan (1985) give an account of the development of sugarcane varieties that starts with Evenson & Kislev (1975) on the relationship between basic and applied science, continues with plant breeding and continues into the international diffusion of genetic material and research capacity.

The productivity gain will be limited by the weakest link in this chain, but from 1961 when data became available, the international system saw public R&D growing at an increasing rate in both DCs and LDCs. This generated substantial yield growth in the DCs and the new varieties could be fairly quickly adapted by the LDCs. There is technology on the shelf for the countries that follow the leaders, and LDCs with good research and extension resources can prosper under these circumstances. However, extension and simple adaptive research lack the glamour of path-breaking new technologies and are often neglected.

Agricultural extension has attracted relatively little attention in the past two decades and there is a serious lack of data for most LDCs. The only compilation is Swanson *et al*. (1991), which does not extend past 1988 and has much missing data. However, Anderson & Feder (2007) estimate the number of extension personnel in the world is about half a million, of which 80 per cent are public sector and 396 000 are in LDCs where they outnumber research scientists by as many as 20 : 1. The regional distribution of workers in the developing countries is in need of updating but, based on Swanson *et al*. (1990, p.56) data on public-sector employees is in table 2.

**Table 2.** LDC extension personnel. Source: Anderson & Feder (2007).

developing region	total extension personnel ('000)
Latin America	28
Middle East-North Africa	34
Asia	277
sub-Saharan Africa	57
total developing countries	396

The balance between R&D and extension has long been an issue, as critics suggested that many of these workers had nothing to extend owing to weak R&D. Also, extension has tended to be the poor relation at the bottom of the funding chain (Thirtle & van Zyl 1994). This resulted in entire budgets being spent on recurrent items like salaries, while there was no fuel for vehicles and hence no farm visits.

Despite these concerns Evenson's (2001) survey of the impact of extension services showed a median IRR of 80 per cent, but with a large variance. This study covered the available work from 1970 to the early 1990s, which fitted production functions with extension as an explanatory variable for output or yields. The problem with this technique, which accounts for most rate of return (ROR) studies on R&D, is that many covariates are omitted and hence the researchers find just what they are looking for: a positive and significant elasticity for extension or R&D. As studies have become more sophisticated, especially by allowing for international spillovers of technology (Schimmelpfennig & Thirtle 1999), RORs have fallen to more reasonable levels of around 30 per cent. Evenson & Pingali (2007) took a different approach, showing that whereas there was correlation between research scientists and the adoption of green revolution modern varieties, there was no correlation with extension officers. Many countries with an abundance of extension personnel did not have a green revolution.

This suggests that the causes of failure and success in extension need to be examined and this challenge was taken up by Feder *et al*. (2001), who suggest there are some generic and universal difficulties in the operation of public extension systems and in the bureaucratic–political environment within which they are budgeted and managed. They find eight factors that can cause deficient performance: the scale and complexity of extension operations; the dependence of success on the broader policy environment; the problems that stem from the less than ideal interaction of extension with the knowledge generation system; the difficulties inherent in tracing extension impact; the profound problems of accountability; the oftentimes weak political commitment and support for public extension; the frequent encumbrance with public duties in addition to those related to knowledge transfer; and the severe difficulties of fiscal unsustainability faced in many countries.

Anderson & Feder (2007) offer solutions to problems with training-and-visit extension, decentralized mechanisms for delivery, fee-for-service and privatized extension, and farmer field schools. Their review emphasizes the efficiency gains that can come from locally decentralized delivery with incentive structures based on largely private provision, much of which will inevitably remain largely publicly funded, especially for impoverished developing countries.

## 3. International comparisons of r&d and productivity

In the 1950s and 1960s science was applied to agriculture in the DCs, with rapidly rising R&D expenditures and productivity growth, whether measured by yields, labour productivity or TFP. During the 1960s and 1970s this process was extended to the LDCs, as the green revolution raised yields, especially in the densely populated countries of Asia. The DCs have good data that have been used to substantiate the claims made above. Obviously, the DCs are responsible for much of the food that is traded, and basic R&D in the DCs should spill over and play a role in LDC productivity. The data for the rest of the world are less detailed, but it is possible to assess the changes in R&D and productivity for both the DCs and the LDCs.

### (a) R&D expenditures in rich and poor nations

**Figure 1.**
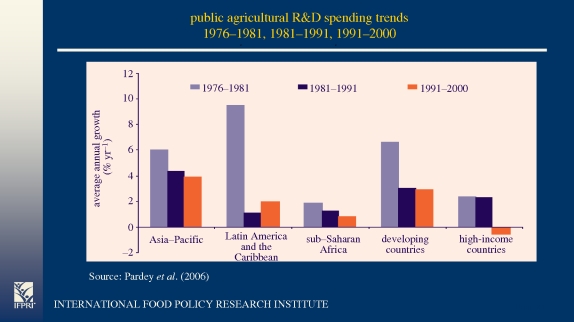
World R&D expenditures by region and income level in 2000 international US$.

In the longer term, supply response depends on the availability of appropriate technology. Public R&D expenditures for the high-income countries fell from 10 534 million constant 2000 international US$ in 1991 to 10 191 million in 2000 (Pardey *et al*. 2006). This fall is minor, but R&D was also retargeted towards public interest areas such as the environment and food safety so the allocation to productivity enhancing research declined substantially (Alston *et al*. 1999). The decline in the high income countries' R&D expenditures is shown in figure 1, which summarizes the world situation. The figure shows that the rate of growth of agricultural R&D expenditures has declined everywhere since the first period.

These regional trends hide a growing divide between the scientific haves and have-nots. In the Asia-Pacific region, just two countries, China and India, accounted for 89 per cent of the $42.5 billion increase in regional spending from 1995 to 2000. Thus, China and India accounted for 59 per cent of the region's scientific spending in 1995, jumping to 73 per cent by 2000. While these huge national agricultural research systems (NARS) are successful, partly because the multinationals will collaborate to gain access to large markets (Pray *et al*. 2007), sub-Saharan Africa (SSA) has suffered from what Lipton & Longhurst (1989) called the Balkanization of research. There are marked returns to scale and the NARS of small countries fare poorly. In SSA the growth rate in full time staff since 1960 has been 4 per cent per annum and 75 per cent have post-graduate training and the remaining 25 per cent have doctorates. The proportion of expatriates has declined from 90 per cent in 1960 to 2 per cent in 2000, which is amazing, but the combination of HIV/AIDS and higher private sector salaries has left many NARS short of staff.

SSA has always lagged so far behind that it is only now showing signs of increased growth. This is partly the result of better policies, institutions and infrastructure, and more robust systems of governance, all of which are allowing the backlog of available technology to be exploited.

### (b) Output, crop yields and animal improvements

The World Development Report (WDR; World Bank 2008) states that developing countries achieved much faster agricultural growth (2.6% a year) than industrial countries (0.9% a year) from 1980 to 2004, accounting for 79 per cent of growth. Their share of world agricultural gross domestic product (GDP) rose from 56 per cent in 1980 to 65 per cent in 2004, with the newly industrializing economies (NIEs) in Asia accounting for two-thirds of the developing world's agricultural growth. The major contributor to growth in Asia, and the developing world in general, was productivity gains rather than expansion of land devoted to agriculture. Cereal yields in East Asia rose by an impressive 2.8 per cent a year in 1961–2004, much more than the 1.8 per cent growth in industrial countries. Only in SSA did area expansion have more impact than growth in yields.

**Figure 2.**
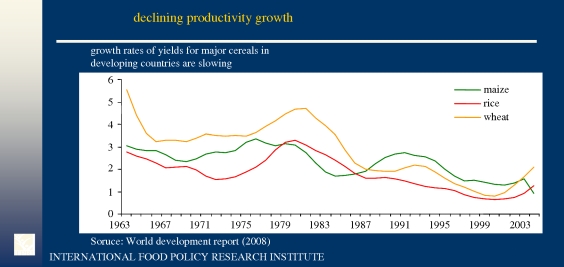
Developing country productivity growth rates for major cereals. Source: World Development Report (WDR; World Bank 2008).

The FAO (2007) and USDA (2009) both report yields for individual crops, measured in physical terms. The World Bank (2007) data are in terms of agricultural value added per hectare, in constant 2000 US$, which obviously allows for enterprise switching and includes all outputs, such as animals and animal products. Figure 2 shows that for the most important cereal crops the growth rates in the developing countries were 3 per cent or better at the height of the green revolution in the early 1980s. Since then growth rates have fallen, so that by 2000 the rates for rice and wheat were about 1 per cent and maize a little better at around 1.5 per cent. There seems to be a slight recovery since 2000, which is surely needed as these growth rates are less than population growth and *per capita* food availability would be falling.

The calculations in this study are split pre- and post-1985 as that was the year at which a downturn in growth rates seemed to begin (table 3). Even so, the results show that all Africa is doing substantially better and South America almost doubling yields in the second period. These positive results need to be set against decline in the other six regions, which reduces LDC yield growth overall from 2.5 to 1.6 per cent. However, this performance is good relative to the DCs, where growth rates have fallen to one-third of their pre-1986 levels and thus the greater decline in the DC's R&D growth is possibly a cause of the greater decline in yield growth.

**Table 3.** Yield growth rates—all grains. Calculated from USDA (2009). Note: these growth rates were measured by regressing the natural logarithm of yield on time. The values marked* indicate growth is not significantly different from zero.

regions	1961–2008	1961–1985	1986–2008
developed countries			
North America	1.834	2.08	2.00
Oceania	0.969	0.65	−0.059*
European Union	1.779	2.86	0.80
former Soviet Union	1.237	1.78	0.518*
Europe (non-EU)	1.485	3.45	0.326*
developed country average	1.46	2.16	0.72
less developed countries			
North Africa	2.079	1.45	2.10
sub-Saharan Africa	0.017*	0.47	0.96
South America	2.218	1.63	3.15
Caribbean	1.43	2.51	0.62
Central America	1.46	2.16	0.40
East Asia	2.86	3.90	1.49
Middle East	1.914	2.13	1.83
South Asia	2.353	2.24	2.15
South-East Asia	1.912	2.08	1.43
less developed countries average	2.10	2.50	1.57

But other factors need to be considered, too. The gains from hybrids and the green revolution-type technologies were exploited earlier in the DCs, so by the second period progress may be more difficult. Note, too, that North America, which is exploiting the major new technology, genetically modified (GM) crops, has no significant fall in yields, whereas the other DCs who are reluctant so far to embrace this science, are doing extremely poorly.

As a general proposition, the low-yield growth in the DCs must mean there will be less technology available to the LDCs in the future, so the falling yield growth in Asia may reflect this. Africa is further behind and has not yet reached this point in the sequence of diffusion. The work on spillovers suggests clear patterns, and within agricultural economics Schimmelpfennig & Thirtle (1999) found there was a cascade effect for the US and the EU. The direction of spillovers was from the US to Northern Europe, from there to Italy and from Italy to the least technologically advanced EU countries such as Greece. This suggests that the lags could be very long. SSA may be able to continue finding technologies for a long time before catching up with Asia, as Asia slows owing to fewer spillovers from the US and Europe. The very large and successful NARS of China, India and Brazil, which benefit from substantial economies of scale, will play an increasing role in technology diffusion.

Productivity in the livestock sector is becoming increasingly important, as with higher incomes the expenditure share of meat, eggs, milk (and fruit and vegetables) rises. Evenson (2001) has shown that productivity growth in the US has always been slow in extensive livestock, but where feed concentrates and selective breeding can be applied, as in pigs and poultry, yield growth can be as fast as in crops. Conradie *et al*. (2009) show that in South Africa, productivity growth in chicken production accounts for the most rapid increases in the Western Cape. Although productivity-enhancing livestock research is not further considered explicitly, it is included in §3*c*, which measures land productivity in value terms, regardless of use.

### (c) Land and labour productivity

The yields above are measured in physical terms, but the *World development indicators* data (World Bank 2007) are published with yields measured in value added per hectare, at constant 2000 US$. This incorporates all outputs and does allow for crop switching, so it may give lower or higher yield growth than the FAO crop level data. It has also been used to estimate labour productivity for Africa and Asia (Thirtle & Piesse 2008). The averages for both land and labour productivity were calculated by regressing the productivity measure on time using a random coefficients model to give an average for the region.

The regional averages are in table 4, starting with the yield for all three groups of developing countries, by continent, which is much the same sample as all the LDCs in table 3. This average yield is lower in the first period at 1.7 per cent, but instead of falling, rises slightly in the second period. Either enterprises other than basic cereals have done better, or switching to higher valued production has contributed to the increase in output value, or both. The Asia average for 1961–2006 of 2.6 per cent is above that for Africa (2%) but this is far higher than expected, given that African agriculture is regarded as failing. Over half the African sample (22 of the 42 countries) had yield growth of over 2 per cent and only eight had less than 1 per cent. Latin America and the Caribbean (LAC) have lower yield growth, but again no decline. Thirtle & Piesse (2008) did not include the DCs, but the last row in this section shows that the former Soviet Union (FSU) and non-EU countries in Europe actually had poorer yield growth than the developing countries.

**Table 4.** Growth in yields and labour productivity using value added data (%). Source: Thirtle & Piesse (2008). All growth rates are significantly different from zero.

region	1961–2003	1961–1985	1985–2003
*yields*			
Africa, Asia, Latin America and Caribbean	1.79	1.70	1.79
Africa and Asia	2.14	1.55	2.27
Africa	2.00	1.37	2.11
Asia	2.60	2.14	2.75
Latin America and Caribbean	1.22	1.27	1.36
FSU and Europe non-EU	0.54	1.94	0.55
*labour productivity*			
Africa	0.40	0.015	0.80
Asia	1.50	1.34	1.56
Latin America and Caribbean	1.35	1.51	1.48
Europe non-EU	3.36	3.00	3.40
EU	4.13	4.93	3.41

The lower part of table 4 shows that yield growth in Asia translated into labour productivity, growing at an average of 1.5 per cent, with only five of the 12 countries having less than 1 per cent growth. For Africa, labour productivity grew at only 0.4 per cent per annum and although the top few countries are in the same league as Asia, almost half the sample (18 countries) actually have negative growth in labour productivity.

This problem has not attracted the attention it deserves. Labour productivity is also crucial to food security because 70 per cent of the poor are rural, and agricultural wages, incomes and poverty reduction are dependent on the productivity of the labour force. The common view is that by 2050 total food output will be sufficient, but there will be extreme insecurity because the poor in SSA will not be producing sufficient food or have enough income to buy it.

There is a clear contrast between the poorest countries and both Europe groups, EU and non-EU, which have rather more than twice as much labour productivity growth. This again follows from the induced innovation hypothesis, as labour is the scarce resource in the DCs. It also fits the view that labour productivity is closely linked to incomes, as Europe, especially the EU member states, is substantially better off.

The poorest countries are all still predominantly agricultural, while the richest have now moved beyond being purely industrial to being economies dominated by the service sector. The greatest empirical regularity in economics is the structural transformation, whereby during the development process agriculture declines in importance relative first to industry and later to services. Labour productivity does not increase dramatically until a country has passed the turning point in the structural transformation, at which the total numbers employed in agriculture start to fall. When countries pass the turning point, their economies change dramatically. Labour productivity in agriculture has to increase rapidly enough for the falling rural and agricultural population to feed the growing urban, industrial labour force. China passed the turning point some years ago and the agricultural population has been declining since 1999. India too has reached the turning point.

These changes have serious implications for the rate and direction of technical change in agriculture. Biological technical change may continue, but it is quite quickly outweighed by mechanical technical change. In China and India, which comprise over 40 per cent of the world population, labour productivity in agriculture will now grow rapidly and so will agricultural incomes.

The problem is that no countries in SSA, except South Africa, have reached the turning point and there seems to be no means of transforming labour incomes at this stage of development. Worse still, labour productivity is a two-edged sword. Inappropriate labour saving technology simply increases the numbers of beggars, as early machinery imports in South Asia showed. However, there are some successes. Herbicide-tolerant GM maize was developed in the US to save labour, but in SSA it has been used with minimum tillage in a way that has reduced erosion and increased area and yields rather than reducing employment (Piesse *et al.* 2009).

### (d) Total factor productivity

Lack of data on input prices prevents the construction of TFP indices using accounting or index number methods. The alternative is programming techniques, such as data envelopment analysis, which allows the use of the Malmquist index. However, this is constructed by comparing each observation with the best practice frontier that is determined from all the observations, so the choice of the peer group changes the index for each country.

The index number alternative is followed by Avila & Evenson (2007), who dealt with the lack of data for share weights by applying values from Brazil and India to all the other LDCs. They apply the share weights calculated for India to Africa and Asia, and those for Brazil to Latin American and the middle income countries. Fuglie (2008) extends this by including China, Indonesia, Japan, the UK and the US as sources of shares, so that the allocation is slightly less crude. For example, the estimates for Brazil were applied to LAC, the Middle East and North Africa (MENA) region and South Africa. The estimates for India were applied to other countries in South Asia as well as SSA except South Africa. The US shares were used for the FSU and the 1967–1990 UK shares (from Thirtle & Bottomley 1992) were used for all of Europe except the USSR.

Fuglie (2008) finds no evidence of a general slowdown in TFP growth from 1970 to 2006. Indeed, the world TFP up to 1989 grew at 0.87 per cent per annum and since 1990 has grown at 1.56 per cent per annum. He also notes that for maize, rice and wheat yield growth fell from 2.29 to 1.35 per cent per annum and output per hectare from 1.96 per cent to 1.95 per cent, while labour productivity growth rose from 1.25 to 1.51 per cent per annum. The major finding that reconciles these results is that it is input growth that has declined, as in the extreme case of the UK, where it was negative. TFP growth largely offset decelerating input growth to keep the real output of global agriculture growing at about 2 per cent per year since the 1960s, but there was a slowdown in the growth of agricultural investment. This is an important finding and explains why the supply response to the food price crisis was so strong. Lack of investment on farms can be corrected far more quickly than lack of new technology where the R&D and diffusion lags are very long.

Fuglie's (2008) results show there is no general decline. Indeed, there is TFP growth that offsets the decline in inputs and keeps output rising. His results are reported by decade. For the DCs and the LDCs, USSR, the FSU, Eastern Europe, LAC, North-East Asia, China, South-East Asia and North America, TFP growth in the 1990s and since 2000 was greater than in the 1980s, which was an improvement on the 1970s. For western Europe, Oceania and SSA the 1990s growth was an improvement on the 1980s, but since 2000 is lower than the 1980s. For the MENA, South Asia and India, both recent periods show lower growth than the 1980s. On average then, his results are heavily biased in favour of growth rather than decline.

Since there is evidence that yield growth may have declined and even labour productivity growth has slowed, it is important to note that TFP is different exactly because it takes account of the land and labour substitutes that are modern intermediate and capital good inputs. Less capital investment and chemicals will increase TFP just the same as more output or higher land or labour productivities.

**Figure 3.**
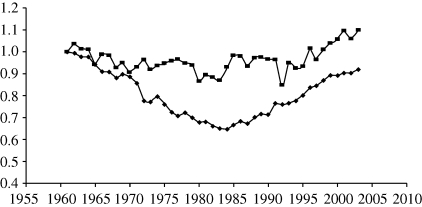
Index of cumulative TFP growth in SSA (1961 = 1; filled black diamonds, sub-Saharan-Africa and filled black squares, not including Nigeria).

Ludena *et al*. (2007) use the Malmquist index to estimate agricultural productivity growth for 116 countries and find that average annual agricultural TFP growth increased from 0.6 per cent to 1.29 per cent between 1961–1980 and 1981–2000. These positive results are used to forecast optimistic increases in productivity to 2040. Most recently, Nin Pratt & Yu (2008) have produced estimates concentrating on SSA using the Malmquist index, but with shares constrained to stay within bounds derived from Avila & Evenson (2007). This is an interesting approach, but it may combine the best or the worst of both alternatives. The results in figure 3 show that TFP was declining in SSA until the mid 1980s, after which there is a dramatic recovery. Up to 1985, TFP declined at 1.67 per cent per annum but then grew at 1.73 per cent, with a further improvement to 1.83 per cent in the 1990s. This is in keeping with earlier Malmquist results, such as Lusigi & Thirtle (1997), although the technique may be inclined to have an upward bias.

**Figure 4.**
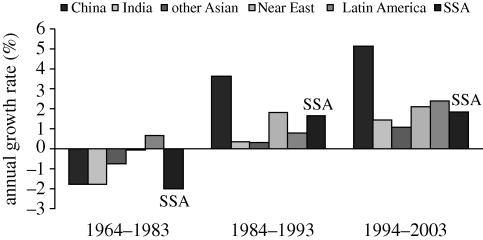
Average TFP growth rate of SSA's agriculture in different periods compared with TFP growth in other regions. Source: Nin Pratt & Yu (2008).

Figure 4 shows TFP growth in SSA relative to Asia and Latin America. The conflict with the index number approach results is clear, as only Latin America had positive growth prior to 1983, and even then it was less than 1 per cent. Then, all the regions improve between 1984 and 1993 and all improve again between 1994 and 2003.

The improvement in SSA is partly owing to better agricultural practices, but it must be stressed that almost all are owing to efficiency increases rather than technological change. Many countries in SSA now have better policies, better governance and improved institutions and infrastructure, which have taken them closer to the technological frontier. However, the frontier is not moving outwards, so the R&D systems are not creating better technologies for the future. Thus, the prospects are limited as the efficiency gains will cease if the frontier is static. So Nin Pratt & Yu (2008) take a pessimistic view in their paper to counterbalance the optimism of Ludena *et al*. (2007).

The above review is sufficient to show that there is no consistent evidence of a decline in TFP. The methods may be imperfect, but all suggest growth rather than decline when using these broad sets of data. Thus, the evidence of decline is confined to TFP studies of individual countries, such as the UK (Thirtle *et al.* 2004), which turns out to be atypical and different even from the rest of the EU. However, the evidence on yields was more dismal and labour productivity is improving very slowly in SSA. This lack of entitlements may well prove to be the least tractable problem.

### (e) Sustainability, maintenance research and environment

Although the literature concentrates on land, labour and TFP, there are also social TFPs, which incorporate changes in environmental quality. From the 1980s in the UK, R&D was redirected away from productivity and towards public interest issues such as the environment, animal welfare and food safety, in line with government policy. The UK disasters in animal production and food safety, such as bovine spongiform encephalopathy (mad cow disease), Creutzfeldt–Jacob disease, *Escherichia coli*, foot and mouth and *Salmonella* suggest that this was required. The FAO argues that animal diseases are now transmitted across borders owing to globalization, and with selective breeding for performance comes higher disease risk, which increases animal health R&D on preventive measures and means more prophylactic pharmaceuticals. For livestock in South Africa, Townsend & Thirtle (2001) showed that maintenance research (animal health) is at least as important as productivity enhancing research and this distinction should also be made in the arable sector, where a substantial share of R&D is to sustain existing yields.

In LDCs, more efficient natural resource use can improve water productivity. For instance, drip irrigation uses scarce water very parsimoniously and is labour-intensive, which suits LDCs with high unemployment. Much is expected of a Gates- and Buffet-funded initiative in which Monsanto and BASF are collaborating with CIMMYT and several NARS to develop water-efficient maize for Africa (WEMA; see www.monsanto.com). The expectation is that by 2020 the project will lead to 2 million extra tonnes of grain and will improve the nutrition of 14–21 million poor people. As climate change raises the incidence of drought, the gains will obviously increase further.

Herbicide-tolerant GM white maize is already being used with ‘planting without ploughing’ in KwaZulu Natal, and is both high yielding and prevents soil erosion (Gouse *et al*. 2006). Soil erosion can also be prevented by labour intensive soil conservation measures, even when population pressure is increasing, as Tiffen *et al*. (1994) showed in their study of the Machakos district of Kenya.

The oil price rises also means expensive fertilizer and will mitigate in favour of precision agriculture to use only the necessary modern inputs. A specific example of what can be done is the encapsulation of sugar beet seeds in the UK with nutrients and plant protection chemicals, which massively reduces input use and pollution as well as giving higher extraction rates (Thirtle 1999). What is needed is soil and water efficient, modern input reducing, low-emission technologies for both DCs and LDCs. Finally, simple improvements in storage technology can prevent heavy post harvest losses and there are health impacts, as GM maize has been shown to have less carcinogenic toxins, while *Bacillus thuringiensis* (Bt) cotton reduces hospital admissions for burns and poisoning from pesticide sprays.

### (f) Precautionary R&D

Some R&D expenditures are targeted at counteracting disasters. As crops become less genetically diverse, the risk posed by new pests or viruses increases. Gene banks are some insurance that other genetic material will be available to counteract the threat. The recent outbreaks of animal diseases suggest that the public resistance to GM crops is irrational. It is the animal production systems that are a danger to health. In the DCs, expenditures on preventive and reactive R&D for mad cow disease, swine flu, foot and mouth and *Salmonella* have increased. In the LDCs, the close proximity of people and animals lies behind the outbreaks of swine and bird flu and their transmission to humans. At present, there is an outbreak of Rift Valley fever in South Africa. Increased animal output and population pressure will mean these outbreaks should be regarded as an increasing threat in future.

## 4. Private dominance, biotechnology and incentives: the demise of the commons

The international system that produced the results outlined above was centred on open access to intellectual property that Dalrymple (2004) called a global public good (GPG), albeit an impure one. This is a reasonable description of the system that was in place from the Second World War to the end of the millennium and it has produced some excellent progress in international productivity growth. However, this is now increasingly under threat.

### (a) Growth of private R&D

Most DC research is now private while over 90 per cent of LDC R&D is public, as shown in table 5. Twenty years ago universities and public laboratories in the DCs did all the basic and strategic research and this created a global commons of intellectual property. Now Monsanto and Syngenta lead and the Consultative Group on International Agricultural Research (CGIAR) and the rest of the international public systems tend to follow.

**Table 5.** Estimated global public & private agricultural R&D, *ca* 2000. Source: Pardey *et al*. (2006).

	expenditures, million 2000 international $			share per cent	
region	public	private	total	public	private
LDCs	12 819	862	13 682	93.7	6.3
DCs	10 191	12 086	22 277	45.7	54.3
total	23 010	12 948	35 958	64.0	36.0

The extent of the domination of the agricultural chemicals, seeds and biotechnology market is apparent in table 6, which is from a survey conducted by USDA. The ‘big six’, which are BASF, Bayer, Syngenta, Dupont, Dow and Monsanto, together spend US$3.6 billion, compared with US$1.42 billion for the other 249 companies operating in these areas and US$4 billion for all the other areas. The total private expenditures on agricultural chemicals is US$2.65 billion and for seeds and biotechnology US$2.37 billion. The effect of this increasing concentration is not clear, but there is an early study of the effect of market structure on innovation in biotechnology (Schimmelpfennig *et al*. 2004).

**Table 6.** Private sector firms and R&D expenditures by type of activity. We thank Keith Fuglie of USDA for this preview of unpublished work.

	number of companies	agricultural R&D in 2006 (billion US$)
agricultural chemical-seed-biotechnology companies	‘big 6’	2.03 + 1.57 (chemicals + seed & biotechnology)
other agricultural chemicals	122	0.62
other seed	82	0.63
other agriculture biotech	45	0.17
farm machinery	35	1.21
animal health	118	1.58
animal and aquaculture genetics	61	0.26
fertilizer	—	0.45
animal feed	—	0.5
total	446	9.02

The several meanings of the term private in the R&D context were discussed by Thirtle & Echeverria (1994). A technology produced by a public R&D institution, funded by public taxes, producing outputs that are in the public domain, is clearly public. Historically, this best describes the invention of biological technologies at the basic end of the science spectrum, where patenting was not possible and a private R&D institution would have no way of appropriating the returns to the investment. At the other extreme John Deere, indulging in adaptive, near-market mechanical innovations is purely private and the returns are secured when the new tractor in which the innovation is embodied is sold at a price that reflects its superiority. Or, alternatively, other companies pay for the use of the patented innovation. Most innovations lie somewhere between these two extremes. Research may be publicly or privately funded, performed by a public or private institution and the innovation produced may be proprietary or in the public domain. Nor is it easy to state a location, as private agricultural R&D is now the province of a few huge multinationals with global reach, noted above. Thus, any simple statement tends to be inaccurate.

Pray *et al*. (2007) start from the fact that private expenditures are now larger than public in most industrialized countries. They outline the history of private research in the developing counties, where plantations (tea, rubber) and processors (tobacco) played a major role. Similarly, haciendas in Latin America were large enough to finance R&D as were sugar plantations such as Lonrho in Africa. Pray *et al*. (2007) estimate that in 1995 private R&D in Asia and Latin America was only 10 per cent of the total, but rising. In SSA it is lower, but not non-existent even in the late 1980s (Thirtle & Echeverria 1994). South Africa is the exception, owing to Monsanto's involvement in maize and cotton. Pray *et al*. (2007) report 525 field trials of GM crops in Africa, as compared with 1235 in Latin America and only 243 in Asia, as of 2003. This is a better indicator than expenditures for South Africa, as Monsanto does most of its R&D in the US. Total global R&D is estimated at US$33-35 billion per annum by the mid 1990s, with a bit over one-third spent by the private sector. Public R&D split about evenly between DCs and LDCs, but 94 per cent of private R&D was in the DCs. However, as noted, the location of the R&D and the use of the innovation can easily be as far apart as California and KwaZulu Natal.

### (b) Biotechnology and incentives

The growing importance of private R&D activity is closely associated with biotechnology and GM crops for two reasons. GM crops account for much of the private sector activity in LDCs and Monsanto is the dominant player, responsible for 39 of the 54 GM events that have been approved for commercial use. The great majority of these involve Bt or herbicide tolerant (or both) soy, maize or cotton. Technically, the development of genetic markers played a key role in moving the public–private boundary as they allowed the identification of specific traits in biological material that were not previously possible. Hence, patenting became possible more widely and the courts pushed the process forward with decisions in favour of patenting. Changes in the US law in 1980 were important as the Diamond *v.* Chakrabarty decision allowed patenting in a case where living organisms were involved (actually an oil eating bacterium that General Electric wanted to patent). Cohen and Boyer were awarded a US patent for their work on recombinant DNA and the Bayh–Dole Act allowed grant recipients, such as universities, to apply for patents on federally funded research. Other DCs followed and in the 1990s the trade-related aspects of intellectual property rights (TRIPS) agreement of the World Trade Organization (WTO) globalized the protection of intellectual property (Wright *et al*. 2007). Many LDCs were unhappy, but had to accept TRIPS in order to keep the advantages of WTO membership.

The technology problem has always been that patents help push the resources committed to R&D towards an optimum, but slow the diffusion of the innovations, given they are frequently non-rival in consumption. Excludability is at odds with maximizing welfare in the case of a non-rival good. Wright *et al*. (2007) compare patents and other means of providing incentives to innovation, such as prizes and contracts. The means of protecting intellectual property rights (IPRs) in agriculture, such as plant patents, plant breeders' rights, utility patents, trade secrets, trademarks and geographical indicators vary in terms of protection levels. There are also alternatives to IPRs, such as hybrid varieties, genetic use restriction technologies, contractually defined rights over tangible property, and mergers and acquisitions. The results are similar in that the trend is towards concentration.

Thus, a consequence of extending patents to plants, in combination with the huge costs of biotechnology research is that the NARS, whose size led to their ascendancy over small private seed companies in the past century, are losing ground to massive multinationals. Herdt *et al*. (2007) note the concentration of varietal development and seed production in less than half a dozen such companies. Biotechnological discoveries and enabling technologies are patented, and since genetic improvement is a derivative process, each incremental improvement adds a further layer of IP constraints. Mergers increase a company's IP portfolio, giving it more freedom to operate and hence an advantage over smaller rivals. The building blocks and the tools all come with IP constraints and are commercially useful only to companies with portfolios covering most inputs. For example, Golden Rice required 40 patents and six material transfer agreements (MTAs). De Janvry *et al*. (2000) report that whereas in 1994, 77 per cent of patents for Bt were held by individuals and independent biotech companies, by 1999 six multinational companies (MNCs) held 67 per cent of Bt patents, 77 per cent of which were by the acquisition of smaller firms.

The level of difficulty in terms of legal costs and simply having any idea of what to patent or otherwise protect is increasingly putting agricultural research beyond the reach of most potential producers. North (1990) explains the importance of institutions that give the correct incentives by pointing out that throughout history, except for the past couple of hundred years and in limited parts of the world, the norm has been rent seeking and directly unproductive activities aimed at redistribution rather than production. Adam Smith's invisible hand is the exception not the rule, and legal activities are almost redistributional by definition. Thus, the scientific revolution in agriculture is in danger of sinking into the mire of rent seeking, with the growth potential snuffed out. So it is Wright *et al*. (2007) who argue that decentralized ownership of IP and high transactions costs can lead to an ‘anti-commons’ phenomenon in which innovations with fragmented IP rights are underused. The evidence is sparse and largely anecdotal, but they do not reject the possibility that IPRs do prevent progress in agricultural technologies, much as they have in pharmaceuticals.

In terms of forecasting, it seems probable that the tangle of IPRs will get worse as time passes. The concentration in technology production is unlikely to be reversed and the international organizations of the CGIAR are likely to finish second to the multinationals. This leaves a vision of a world of oligopolistic competition or collusion in agricultural innovations. The objective functions of Monsanto and its competitors may not be totally incompatible with that of the CGIAR, but they are surely not the same. Any MNC has to protect its own position, make profits and satisfy its shareholders, whereas the CGIAR should have poverty reduction and adequate nutrition for the poorest at the top of its agenda. Since there is a strong negative correlation between profitability and ending poverty, it is unlikely that the MNCs can be at the forefront of poverty reduction.

## 5. Estimates of the agricultural investments needed to end hunger

By 2050, the world population is expected to grow by 40 per cent (from 6.5 to 9.1 billion) and allowing for increased incomes and changes in diet, global demand for food, feed and fibre is expected to grow by 50 per cent by 2030 and 70 per cent by 2050 (Bruinsma 2009). There are a wide range of estimates of demand and supply, but most consider that although demand can be met, some intervention will be required to ensure that supply keeps up and thus prices rises are prevented. More will be required to reduce poverty and move towards the FAO's stated aim of ending world hunger by 2050.

The conventional wisdom that the very high RORs to agricultural R&D indicate underinvestment implies that increasing investment is an economically efficient way of increasing food output and decreasing hunger. Beintema & Elliot (2009) suggest that the current yield growth rate of about 1 per cent can be increased to its historical level of about 2 per cent by increasing investments in the LDCs. The estimate in this paper is that the compound growth rate needed to increase output by 70 per cent over the next 40 years is 1.34 per cent per annum. If the output elasticity of agricultural R&D is as low as 0.05, which is the bottom end of the range of estimates (see Nin Pratt & Fan (2009), discussed below), then this crude approach suggests that with a US$36 000 million world total for R&D, a 6.8 per cent increase would take growth from 1 per cent to 1.34 per cent and this would cost about US$2500 million per annum. Von Braun *et al*. (2008) use a lower estimate of current output growth, 0.53 per cent per annum, and calculate that an extra US$5000 million of R&D investment in the LDC NARs and the CGIAR would be needed to raise this to 1.55 per cent per annum. Using the more pessimistic R&D elasticity and without the targeting of expenditures, a 20 per cent increase of US$7200 million extra R&D investment would be needed to increase output growth by 1 per cent per annum. These figures give a good indication of the magnitudes involved, against which other estimates can be judged.

Tweeten & Thompson (2008) estimate that cereal demand will grow at 1.17 per cent per annum, giving an increase of 79 per cent by 2050. With linear yield growth projected to be 1.07 per cent per annum, giving 71 per cent more output implies excess demand at current prices and they estimate that real prices would rise by 44 per cent. They assume no area expansion, so all the increase in output must come from yields. Estimates by Rosegrant *et al*. (2008) show that cereal demand increased by 0.9 per cent per annum, giving a total increase of 56 per cent, or 1.048 billion tons. After allowing for expansion of planting, and irrigation offset by an average diversion to biofuels, their yield gain estimates are 14 per cent lower, resulting in excess demand that increases rice prices by 60 per cent and wheat and maize by over 90 per cent (from Fisher *et al*. 2009). To prevent this outcome Rosegrant *et al*. (2008) estimate that a 13 per cent per annum increase in public investment, especially in R&D, would produce a 0.4 per cent per annum increase in output, which would lower prices enough to halve the number of malnourished children.

Under reasonable assumptions on R&D elasticities, von Braun *et al*. (2008) calculate the impact of LDC investment in R&D doubling from nearly 5000 million 2005 US$ per annum to nearly 10 000 million 2005 US$ by 2013. In the first scenario, where investments are allocated to maximize output (by equalizing the marginal return to R&D across regions), the impact of seven years of doubled R&D (US$35 000 million total) is a 1 per cent increase in output by 2020 and a reduction of the number of people in $1 per day poverty of 203 million. The bulk of the increased R&D is allocated to East, South-East and South Asia, which have the highest R&D elasticities. If poverty reduction is the target, much more is allocated to SSA and South Asia and by 2020 output is increased by 0.58 per cent, but the number taken out of $1 per day poverty is 282 million. In SSA 144 million would be taken out of $1 per day poverty, practically halving the poverty rate, from 48 per cent to 25 per cent. For South Asia, the equivalent figure is 124 million, with the poverty rate reduced from 35 per cent to 26 per cent.

This work is an early report from an ambitious ongoing project on ‘best bet’ programmes to be scaled up in the future strategy of the CGIAR institutions (von Braun *et al*. 2009). The latest estimate on the R&D requirements is Nin Pratt & Fan (2009) who have refined their earlier work. They review the evidence on R&D and poverty elasticities and determine the required investment in LDC NARS and the CGIAR and its regional allocation, in order to maximize output growth or minimize poverty. They show that to maximize output growth, R&D investment should be mainly in South-East and South Asia, but if poverty reduction is the aim then SSA and South Asia should be particularly targeted.

## 6. Conclusions

This paper investigates the productivity slowdown in the DCs and its impact on the prospects for productivity growth in the LDCs. It questions the existence of such a slowdown, pointing out that although yield growth has slowed in aggregate, labour productivity growth varies and TFP has improved in most regions. To the extent that there is a break in the trends for all measures, it comes in the mid 1980s, which fits with the new FAO data showing that the long fall in food prices practically ceased from this time (Piesse & Thirtle 2009).

The paper stresses the interactions between DCs and LDCs, between public and private R&D and between sectors, as countries such as China and India have reached the transitional stage where industry and urbanization have taken the leading role in the growth process. Similarly, agriculture not only reacts to the oil price because of fertilizer, fuel and transport costs, but has become a part of the energy industry, owing to the rapidly growing demand for biofuels. This is a challenge and opportunity for the LDCs, as well as a threat.

Although the food price crisis generated pleas for increased funding, the economic downturn will mean that few donors will increase funding for public R&D. So, Monsanto and the other multinationals will continue to account for a growing share of total R&D funding. Thus, the gene revolution will lack the public sector agenda that led to the green revolution having a poverty focus. Private companies must operate where profits can be made and this precludes the least resource rich, the most marginal and the most distant farming areas. The prospects for growth may well be better than those for poverty reduction. The public sector and the international institutions increasingly have to find a way through the growing tangle of IPRs that threaten what used to be a GPG. This is unfortunate, as in the past agricultural productivity has been an important source of poverty reduction as it helps the rural poor increase their welfare directly and also helps the urban poor by lowering food prices. Perhaps the reform of the CGIAR may help alleviate this potential loss by extending the role its institutions play in spreading the green revolution (Pingali & Kelley 2007). These problems will be exacerbated by climate change, as both higher temperatures and a rise in sea levels will hit tropical LDCs hardest.

Against this gloom, there is the fact that SSA is finally making slow progress, so that some of the poorest are likely to benefit. However, it is probably not possible to generate sufficient food output or incomes in much of SSA to feed the population at all adequately. Higher up the income distribution, more countries are reaching the turning point in the structural transformation, when agricultural labour productivity rises as a result of labour being withdrawn from agriculture. Agriculture has to provide both food and labour for industrialization. If it succeeds it transforms itself and the country joins the ranks of the industrialized, urbanized group with greater prosperity. If it fails, it holds back industrialization and urbanization and slows the development process. So, for LDCs at all levels, there are prospects of productivity growth, but those with very little technological capacity will be disadvantaged. Increasing labour productivity is the norm for the industrializing countries, but it is SSA where both labour productivity growth and employment need to increase, and this is where biggest regional challenge lies. Increasing yields will also be needed if agriculture is to meet world demand for both food and energy. This competition for agricultural output will re-emerge as the recession eases and China and India resume their rapid growth and transformation.

## Glossary

Biofuels: the use of agricultural output for ethanol and biodiesel.
Biological technical change: yield enhancing technologies that increase the fertilizer responsiveness of plants and thus increase yields.
Data envelopment analysis: a non-parametric approach to constructing efficiency frontiers using linear programming techniques.
Elasticity: in this paper, the proportional change in output resulting from a proportional increase in R&D. Using proportional changes makes the elasticity a pure number; it does not vary with units of measurement and is comparable across studies.
Golden rice: vitamin E enhanced rice developed by the Rockerfeller Foundation.
Green revolution: general term for increased yields due to improved plant varieties introduced from the 1960s, especially in Asia.
Index number techniques: index number theory is used in the accounting approach to measuring total factor productivity, by construction rather than estimation.
Internal rate of return: the rate of return on investments in R&D, found by discounting the value of future output increases.
Land productivity: a partial productivity measure, in that it is with respect to only one of several inputs. Used in this paper to mean the value of output per unit of land, as opposed to crop yield, which is measured in physical terms. This allows for enterprise switching, say from low value crops to high value activities like poultry.
Labour productivity: the other most common partial productivity measure, which is output per unit of labour, or the average product of labour.
Malmquist index: an index of total factor productivity derived from the DEA approach, which does not require input prices.
Marginal productivity: the increment in output gained when an input is increased by one unit. Most importantly, in a competitive market, the wage is equal to the marginal product of the last unit of labour hired.
Marginal return: the return to the last or marginal unit of an input.
Mechanical technical change: the substitution of machinery and equipment for labour, which has transformed labour productivity in the developed countries.
Production function: the relationship between output and the inputs used to produce it.
Random coefficients: estimation method developed by Swamy and used for panel data. The groups are estimated as separate entities and the final coefficients are the group means. This is used to generate land and labour productivities for Africa and Asia, where it is an average of the individual country results.
Rent seeking: economic activity that does not increase output. Such activities are simply redistributing the pie to the advantage of the protagonist and are therefore also known as directly unproductive activities.
Returns to R&D: the increases in output or productivity that can be attributed to R&D expenditures.
Supply response: the increase in output resulting from an increase in the output price, which is usually measured as an elasticity.
Total factor productivity: output per unit of all inputs, appropriately weighted to account for their relative importance.
